# Antimicrobial Effect of Nano-Calcium Hydroxide on the Four- and Six-Week-Old Intra-Canal *Enterococcus Faecalis* Biofilm

**DOI:** 10.30476/dentjods.2022.90215.1476

**Published:** 2023-06-01

**Authors:** Mohammad Frough Reyhani, Negin Ghasemi, Amin Salem Milani, Masoumeh Abbasi Asl

**Affiliations:** 1 Dept. of Endodontics, Dental Faculty, Tabriz University of Medical Sciences, Tabriz, Iran; 2 Dept. of Endodontics, Dental and Periodontal Research Center, Dental Faculty, Tabriz University of Medical Sciences, Tabriz, Iran; 3 Postgraduate Student, Dept. of Endodontics, Dental Faculty, Tabriz University of Medical Sciences, Tabriz, Iran

**Keywords:** Biofilm, *Enterococcus faecalis*, Nano-calcium hydroxide

## Abstract

**Statement of the Problem:**

*Enterococcus faecalis* (*E. faecalis*) is one of the most important microorganisms in the evaluation of the antibacterial effects of intra-canal medications due to its ability to penetrate dentinal tubules and form biofilms. Calcium hydroxide, as the most common intra-canal medication, has little effect on this bacterial species. In contrast, it is hypothesized that nano scale hydroxide particles are more effective due to their smaller size and higher surface-to-volume ratio.

**Purpose:**

This study aimed to investigate the antimicrobial effect of nano-calcium hydroxide on the four- and six-week-old intra-canal *E. faecalis* biofilms.

**Materials and Method:**

In this *in vitro* study, seventy maxillary single-canal premolar teeth were used. After cleaning and preparing the root canals, the samples were placed in vials containing *E. faecalis* solution in which the culture medium was changed daily. Each group was divided into three subgroups (n=20) in terms of the antimicrobial material used as the intra-canal medication including subgroup 1: nano-calcium hydroxide, subgroup 2: calcium hydroxide, and subgroup 3: phosphate-buffered saline solution (control group). The antimicrobial property was measured by counting colony-forming units (CFU). The data were analyzed with Mann-Whitney U and Kruskal–Wallis tests. Statistical significance was set at *p*< 0.05.

**Results:**

The mean CFU in the six-week-old biofilm group was significantly higher than that in the four-week-old biofilm (*p*= 0.003). A comparison between the subgroups showed a significant decrease in CFU in the six-week-old biofilm in the nano-calcium hydroxide subgroup compared to that in the calcium hydroxide subgroup (*p*= 0.002). However, the decrease was not significant in the four-week-old biofilm group (*p*= 0.06).

**Conclusion:**

Under the limitations of the present study, the antimicrobial properties of nano-calcium hydroxide were higher than conventional calcium hydroxide on mature biofilm, whereas the antimicrobial properties were not clinically and significantly different on immature biofilm.

## Introduction

*Enterococcus faecalis* (*E. faecalis*) is a facultative anaerobic gram-positive microorganism and one of the most resistant strains within the root canal due to its different virulence factors [ 1 ]. It is resistant to most intra-canal disinfection procedures; therefore, it is the most common microbial species in root canal treatment failures [ 2 - 3 ]. It can also tolerate harsh environments, including high pH (such as calcium hydroxide), dryness, and high salt concentrations [ 1 ]. The ability of *E. faecalis* to penetrate dentinal tubules enables it to evade the effect of endodontic instruments and irrigation solutions [ 4 ]. *E. faecalis* can invade up to a depth of 50‒300 µm inside the dentinal tubules [ 5 ].

One of the most prominent features of this bacterial species is its ability to form biofilms. A biofilm is an organized bacterial aggregation that is resistant to antimicrobial agents compared to the planktonic state, and over time, the structure of the biofilm becomes more organized with increasing resistance [ 6 ]. Eventually, mineralization and calcification occur in the biofilm structure, which is an indication of its maturity. In the case of the *E. faecalis* biofilm, this time corresponds to the sixth week of evolution. Biofilms under 6 weeks old are known as young biofilms, and those over 6 weeks old are referred to as mature biofilms [ 6 - 7 ]. As the *E. faecalis* biofilm matures, the biofilm structure gradually calcifies, making it more difficult to eliminate this mature calcified biofilm through conventional methods, eventually leading to persistent root canal infections [ 8 ]. It has been reported that after six weeks, the signs of complete mineralization and maturation are observed in the *E. faecalis* biofilm structure [ 4 ]. Therefore, a six-week *E. faecalis* biofilm developmental period is considered as the temporal index for mature biofilm. Most previous studies have been performed on young biofilms; however, in most cases, root canal biofilm is several weeks or months old at the time of root canal treatment [ 7 , 9 ].

Recently one study have been carried out to introduce a material with better properties that can overcome the resistance of bacteria to antibiotics, such as the use of nanotechnology in the nano-calcium hydroxide [ 10 ], by changing the calcium hydroxide particle size. The conventional calcium hydroxide particles range from 1 to 10 µm in size [ 11 ], whereas the dentinal tubules’ diameter is approximately 2‒2.5µm near the pulp. Consequently, the conventional calcium hydroxide particles, which are larger, cannot easily penetrate the dentinal tubules [ 12 ]. An increase in the surface-to-volume ratio of the particles increases the solubility and chemical and antibacterial activities of antibacterial agents in the root canal [ 13 ]. Studies have shown that the smaller the nanoparticles and the higher their concentration is, the higher their antibacterial activity will be [ 14 - 15 ]. Previous studies using dentin block removal and agar diffusion methods have shown that the antibacterial properties of nano-calcium hydroxide are higher in the planktonic stage of *E. faecalis* compared to that in calcium hydroxide [ 16 - 17 ]. In addition, using nano-calcium hydroxide as an intra-canal medication for one week and one month resulted in a lower reduction in resistance to root fracture compared to calcium hydroxide [ 10 , 18 ]. This study aimed to investigate the effect of antimicrobial nano-calcium hydroxide on the 4- and 6-week-old *E. faecalis* biofilms compared to conventional calcium hydroxide.

## Materials and Method

In this *in vitro* study, the maxillary single-rooted and single-canal premolar teeth were used. The teeth had been extracted for orthodontic purposes. Patients’ informed consents were obtained. The teeth had fully developed apex and had no caries, cracks, fractures, and previous endodontic treatments. This study was approved by the Ethics Committee of Tabriz University of Medical Sciences under the code IR.TBZMED.REC. 1397.622.

The periodontal tissues and calculi were eliminated from the sample surfaces using an ultrasonic device (Cavitron, Dentsplx Ltd, Wexbridge, UK), and the samples were stored in 0.5 % chloramine T solution until the study was instituted. The tooth crowns were separated from the roots at DEJ using a diamond disk (Leica, SP 1600 Microtome, Nu Block, Germany) under water cooling spray to achieve a standard length of 12 mm.

After determining the working length with #15 K-file (Dentsplx. Maillefer, Ballaigues, Switzerland), the root canals were debrided and shaped using the RaCe Rotary System (FKG, La Chaux de-Fonds, Switzerland), and the #40, 4% taper RaCe file was used for the apical preparation. The irrigation solution used during instrumentation was 2.5% NaOCl, which was carried out with a 10-mL syringe with a 30-gauge needle. To eliminate the smear layer, 1 mL of 5.25% NaOCl solution was used, followed by 1 mL of 17% EDTA solution for 1 and 3 minutes, respectively. The final irrigation was carried out with normal saline solution. The samples were grouped based on the age of the biofilm formed within the root canals as group A with 4-week-old biofilm and group B with 6-week-old biofilm.

After preparing the root canals, the samples were autoclaved for 20 minutes at 121ºC and 15 psi to eliminate all the existing microorganisms. Pure bacterial culture (ATCC 29212) was incubated at BHIB and 37ºC under 10% CO_2_ pressure for 24 hours, and a solution was prepared to form *E. faecalis* biofilms, containing 10^8^ bacterial cells/mL of bacterial cells, equivalent to an OD=1 (optical density) of the UV spectrophotometer device. The teeth were placed within sterile vials, and 1 mL of the microbial suspension was added to each vial. A new culture medium was added to the vial every day. The teeth were placed in sterile micro-tubes containing 1 mL of the microbial suspension.

**Figure 1:**
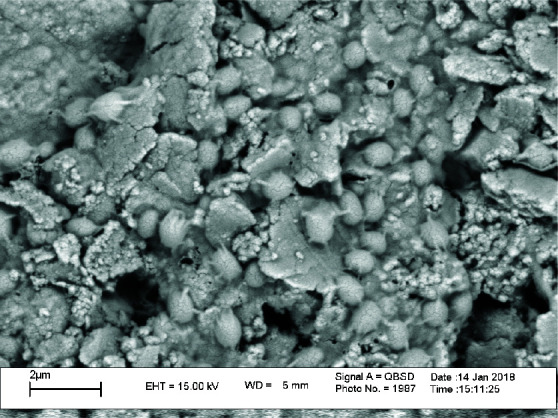
Scanning electron microscope analysis of *E. faecalis* biofilm development in 6 weeks on root canal dentin surface (2000× magnification)

In the group A, the procedure lasted for four weeks, with six weeks in the group B. Subsequently, five samples from each group were randomly selected, cut longitudinally, and examined under an electron microscope (MRIA 3-FEG-SEM-Tescan, Brno, Czech), to ensure that biofilms had formed (Figure 1).

Each group was randomly divided into three subgroups (n=20) in terms of the antimicrobial agent used including subgroup 1, in which nano-calcium hydroxide (Pulpdent Corporation, USA) was placed in the canal using a lentulo spiral and distilled water used as vehicle, subgroup 2 in which calcium hydroxide (Pulpdent Corporation, USA) was placed in the canal using a lentulo spiral, and subgroup 3 (PBS) which was used as a control and the canal was only irrigated with 2mL of phosphate-buffered solution. 

After one week, the antimicrobial agent was removed from the canals with 10 mL of distilled water and dried with a paper point. To collect dental debris, #4 and #5 Gates-Glidden drills (Dentsply, Maillefer, Ballaigues, Switzerland) were used. Dentin chips were weighed with a sensitive electronic weighing machine (ASD CO, LTD, Japan) and 10 mg of dentin chips were weighed for each sample. Then the chips were placed in sterile tubes and 2 mg of physiologic serum was added to each tube and mixed for 20 seconds. Ten-folded serial dilutions were made up to a concentration of 10-7. The debris collected from each tooth was separately placed in new sterile micro-tubes.

To each micro-tube containing the debris (in three subgroups of calcium hydroxide, nano-calcium hydroxide, and phosphate-buffered saline solution), 500μL of sterile distilled water was added. Then, 25mL were taken from each micro-tube and cultured separately on plates containing the Mueller-Hinton *agar* (MHA) culture medium. After incubating for 24‒48 hours at 37ºC, the plates were examined, and the colony counts were determined. Since the evaluated volume for counting was $\frac{1}{20}$ of the total volume of the sample, the counted colony number was multiplied by 20.

The results were reported using descriptive statistics (mean±standard deviation) to determine the antimicrobial properties in the three antimicrobials subgroups. The *Kruskal-Wallis* test was used due to the non-normal distribution of the data to compare the CFUs in the three nano-calcium hydroxide, calcium hydroxide, and phosphate-buffered saline solution (PBS) subgroups at 4- and 6-week intervals separately. A comparison between CFUs at 4- and 6-week intervals were performed separately in each group using the Mann-Whitney U test due to the non-normal distribution of the data. The analysis was performed using SPSS 17. The significance level was set at *p*< 0.05.

## Results

Table 1 and Figure 1 present the means and standard deviations of CFU variable in the two groups and at the two intervals. The results showed the nano-calcium hydroxide had the least amount of CFU variable in 4 weeks and 6 weeks interval.

**Table 1:** The mean and standard deviation of colony-forming units (CFU) variable in the two groups and the two intervals

Evaluation time	Antimicrobial agent	Mean	SD*
4 weeks	Nano-calcium hydroxide	282.0000	188.79501
	Calcium hydroxide	561.0000	656.97032
	PBS**	5005.5000	3.02765
6 weeks	Nano-calcium hydroxide	89.0000	56.26327
	Calcium hydroxide	333.0000	337.64051
	PBS	5505.5000	3.02765

* **SD:** Standard deviation

** **PBS:** Phosphate-buffered saline solution

## Discussion

This study aimed to investigate the antibacterial effect of nano-calcium hydroxide on the 4- and 6-week-old *E. faecalis* biofilm, compared to calcium hydroxide. The results showed the significant impact of nano-calcium hydroxide in eliminating the biofilms at both biofilm maturation stages, with a more significant effect on old biofilm. 

One of the factors affecting the success of root canal treatment is the maximum elimination of bacteria from the root canal system before obturation, which is difficult to achieve due to resistant microorganisms, such as *E. faecalis* in failed root canal treatment cases [ 19 - 20 ]. Therefore, various materials with antibacterial activities have been studied on this bacterial species, and nanotechnology is an area of interest in this respect [ 21 ]. The high ability of *E. faecalis* to invade dentinal tubules and form biofilms has prompted investigations into the antimicrobial activities of materials on the biofilm because it is more difficult to eliminate biofilms compared to planktonic bacteria due to their organized structure [ 22 - 23 ]. On the other hand, periapical periodontitis is a disease associated with biofilm [ 4 ]. Accordingly, in this study, the two immature and mature forms of *E. faecalis* biofilms were used.

Calcium hydroxide is the most commonly used intracanal medication [ 24 ]. Its main features are tissue solubility and high pH, which are fatal for most microorganisms within the root canal [ 25 ]. Hydroxyl ions resulting from its reaction with water destroy the cell wall and the cell’s protein structure [ 12 - 13 , 26 ]. Calcium hydroxide requires at least one week to reach to a pH of 12. It is shown that at a pH of 11.5, *E. faecalis* cannot proliferate. However, a ten-day period for calcium hydroxide use does not affect this bacterial species [ 13 ]. The proton pump in this bacterial species cell wall, the dentin buffering capacity in the clinical samples, the bacteria’s deep penetration into dentin tubules, and the biofilm formation characterize the behavior of this bacterial species in the face of calcium hydroxide. Conventional calcium hydroxide particles range from 1 to 10 µm in size, whereas the dentinal tubules’ diameter is approximately 2–2.5µm near the pulp; therefore, the conventional calcium hydroxide cannot penetrate well into dentinal tubules [ 12 ]. However, nanoparticles can penetrate better due to their small size and are more likely to persist within the dentinal tubules. Zand *et al*. [ 18 ] showed that the penetration depth of calcium hydroxide nanoparticle in all three regions of the root was significantly higher compared to conventional calcium hydroxide particles. In both groups, the penetration depth increased from the apical to the coronal region. High levels of surface-to-volume ratio in nanoparticles and their ion density result in their interaction with the environment, making them highly capable of antibacterial activity [ 17 ]. Kishen *et al*. [ 8 ] attributed the increase in the nanoparticles’ antibacterial efficacy during their use in root canal treatment to the electrostatic binding between these materials and the dentin wall. These results were consistent with those reported by Dianat *et al*. [ 13 ] findings concerning higher antibacterial properties in the nanoparticles.

In the present study, the medications were preserved in the root canals for seven days; an increase in this duration is suggested in future studies. The extracted teeth were used to simulate the clinical condition. After the culturing and biofilm formation within the root canal, the antimicrobial agents were exposed to bacterial biofilms to evaluate the dentin inhibitory effect on the materials’ antimicrobial ability.

The method for determining the antimicrobial activity was CFU counting in the present study; however, in previous studies, mostly agar diffusion technique has been used, which is less accurate than CFU counting [ 27 ]. Culture medium-based studies have estimated the *E. faecalis* prevalence in the apical periodontitis at 37%; however, it was 77% in the PCR technique [ 28 ]. Therefore, it is suggested that this method be used for a more accurate determination of the nano-calcium hydroxide antibacterial properties as an intra-canal medication in future studies.

Using normal saline for eliminating calcium hydroxide was one of limitations of this study. It is not possible to be sure that some remnants of calcium hydroxide were not included in the collected debris, which should be considered in future studies.

## Conclusion

The results showed the significant impact of nano-calcium hydroxide in eliminating the biofilms at both biofilm maturation stages, with a more significant effect on old biofilm.

## Acknowledgement

The authors wish to thanks dental and periodontal research center of Tabriz dental faculty for their supports. 

## Conflict of Interest

The authors declare that they have no conflict of interest.
